# Unraveling the metabolic landscape of *Exophiala spinifera* strain FM: Model reconstruction, insights into biodesulfurization and beyond

**DOI:** 10.1371/journal.pone.0317796

**Published:** 2025-01-29

**Authors:** Hamta Babaei Naeij, Zahra Etemadifar, John Kilbane, Mohammad Hossein Karimi-Jafari, Sepideh Mofidifar

**Affiliations:** 1 Department of Cell and Molecular Biology & Microbiology, Faculty of Biological Science and Technology, University of Isfahan, Isfahan, Iran; 2 Division of Biology, Illinois Institute of Technology, Chicago, IL, United States of America; 3 Department of Bioinformatics, Institute of Biochemistry and Biophysics, University of Tehran, Tehran, Iran; Federal University Dutse, NIGERIA

## Abstract

*Exophiala spinifera* strain FM, a black yeast and melanized ascomycete, shows potential for oil biodesulfurization by utilizing dibenzothiophene (DBT) as its sole sulfur source. However, the specific pathway and enzymes involved in this process remain unclear due to limited genome sequencing and metabolic understanding of *E*. *spinifera*. In this study, we sequenced the complete genome of *E*. *spinifera* FM to construct the first genome-scale metabolic model (GSMM) for this organism. Through bioinformatics analysis, we identified genes potentially involved in DBT desulfurization and degradation pathways for hazardous pollutants. We focused on understanding the cost associated with metabolites in sulfur assimilation pathway to assess economic feasibility, optimize resource allocation, and guide metabolic engineering and process design. To overcome knowledge gaps, we developed a genome-scale model for *E*. *spinifera*, iEsp1694, enabling a comprehensive investigation into its metabolism. The model was rigorously validated against growth phenotypes and gene essentiality data. Through shadow price analysis, we identified costly metabolites such as 3’-phospho-5’-adenylyl sulfate, 5’-adenylyl sulfate, and choline sulfate when DBT was used as the sulfur source. iEsp1694 encompasses the degradation of aromatic compounds, which serves as a crucial first step in comprehending the pan metabolic capabilities of this strain.

## Introduction

Fuel oil consumption has remained a prominent energy source, and the demand for oil continues to escalate in response to rapid population growth. However, it is crucial to recognize that oil pollutants present significant safety hazards, which lead to a substantial increase in treatment costs [1]. Air pollution is a major environmental concern in several developing countries that experience industrialization. Sulfur dioxide, emitted by coal-fired power plants, is the main pollutant that causes haze formation, which results in one million premature deaths every year [2]. The increasing environmental concerns have driven the need to remove sulfur-containing compounds from fossil fuels. Biodesulfurization can be used as a complementary method of hydrodesulfurization, the prevalent method of petroleum desulfurization in refineries [3]. Many studies have been carried out to develop biological desulfurization of polyaromatic sulfur heterocycles (PASHs), like dibenzothiophene (DBT), with bacterial biocatalysts [4–6]. However, fungi are known for their metabolic versatility and are expected to have the capacity to assimilate a wide range of sulfur sources [7]. They are capable of metabolizing a wide range of aromatic hydrocarbons through cytochrome P450 and their extracellular enzymes [8, 9].

The fungal genus *Exophiala*, comprising black yeast anamorphs, melanized Ascomycete, is known for highly polymorphic life cycles and remarkable dual ecology. Numerous species exhibit significant human-pathogenic potential [10, 11] while others are known as polyextremotolerant microorganisms with extremotolerance to acidic pH, radiation, oxidative stress, toxic heavy metals and harmful aromatic compounds [12]. Some *Exophiala* species have unique ability to thrive in environments enriched by toxic hydrocarbons such as benzene, toluene and xylene [12, 13]. As a case in point, *Exophiala spinifera* strain FM isolated from oil-contaminated soil, is capable of utilizing 99% of DBT(0.3 mM) as sole sulfur source by co-metabolism reaction with other carbon sources [14]. GC–MS and HPLC techniques showed that this strain produced 2-hydroxy biphenyl, a sulfur free compound that is converted to 1,3-benzenediol, 5-hexyl during 7 days of incubation at 30°C and 180 rpm shaking [14]. It might be concluded this strain desulfurize DBT by passing through a way similar to 4S pathway, an aerobic desulfurization pathway that converts dibenzothiophene (DBT) into 2-hydroxybiphenyl and sulfite [6, 15–18], making it a potential candidate for use in bioremediation efforts.

Studying the mechanisms by which *E*. *spinifera* is able to survive and thrive in such challenging conditions may provide insights into the evolution and adaptation of fungi to extreme environments. However, despite numerous efforts, desirable desulfurization rates are yet to be attained likely due to the fact that most of studies have solely targeted the *dsz* genes, coding enzymes involved in desulfurization of DBT via the 4S pathway [19, 20]. Given that cellular phenotypes are the manifestations of complex interactions among various gene products and environmental factors, a systems biology approach is useful for studying desulfurization process [17].

The availability of the whole genome sequencing technologies presents an opportunity to study the native potentials of *E*. *spinifera* strain FM at the system level. Genome-scale metabolic models (GSMMs) can be used as a bottom-up systems biology tool to connect genes, proteins, and reactions, enabling metabolic and phenotypic predictions based on specified constraints [21]. GSMM reconstructions are useful knowledge-bases for numerous applications, including prediction of enzyme functions [22], comparative analysis between closely related species [21, 23–25], improving antibiotic production [26] and metabolic engineering, for instance, predicting gene modification strategies to overproduce desired compounds accelerating the process [27]. Moreover, GSMMs that incorporate sequencing, biochemical, physiological, and ‘omics’ data, represent valuable organism-specific databases to obtain a better understanding of the physiological response of a given microorganism towards different milieu conditions. This enables the development of cost-effective bioremediation procedures that surpass current techniques [28]. By understanding the pollutant degradation capability and survival of *E*. *spinifera* [21, 29, 30], we can develop cost-effective biodesulfurization procedures to enhance the ability of desulfurization in this strain.

In recent decades, genome-wide reconstructions of metabolism have been produced for a plethora of model organisms, spanning from bacteria to higher eukaryotes [31, 32]. Well-known organisms like *Saccharomyces cerevisiae* have been studied more often and in greater detail than other organisms to date [21, 33]. However, less extensive efforts have been dedicated to other so-called non-conventional or non-Saccharomyces yeast species, despite their relevance for biotechnological applications, as well as for basic and biomedical research. It is important to note that yeast biodiversity is vast and extends beyond the models reconstructed up to now.

This study aims to perform a comprehensive bioinformatics analysis of the whole genome sequence of *E*. *spinifera* strain FM, with the ultimate goal of reconstructing the first *in silico* metabolic model for this organism. Bioinformatics analysis involved the use of state-of-the-art tools and techniques to identify genes and pathways that are involved in the metabolic processes of catabolizing sulfur-containing xenobiotics and other metabolic capabilities in this strain. We validate the model using the available desulfurization and growth data in the literature and use it to study the effects of various medium components, such as carbon sources on desulfurization activity of *E*. *spinifera*. We assess the properties of the metabolic network such as flexibility and robustness, using Flux Balance Analysis (FBA), shadow price analysis and gene essentiality analysis. The reconstructed metabolic model covers the key metabolic pathways, such as central metabolism, amino acids biosynthesis, nucleotide metabolism, and sulfur metabolism, that describe the assimilation of sulfur into biomass and provide a detailed and comprehensive understanding of the biochemical pathways and networks that underlie the xenobiotic metabolism of this organism, and will serve as a valuable resource for future studies aimed at understanding the biology of this important strain, and unveiling how to better exploit its nature for industrial purposes, and suggesting further model-driven hypotheses.

## Results

### Genome sequencing and assembly

**Fig 1:**
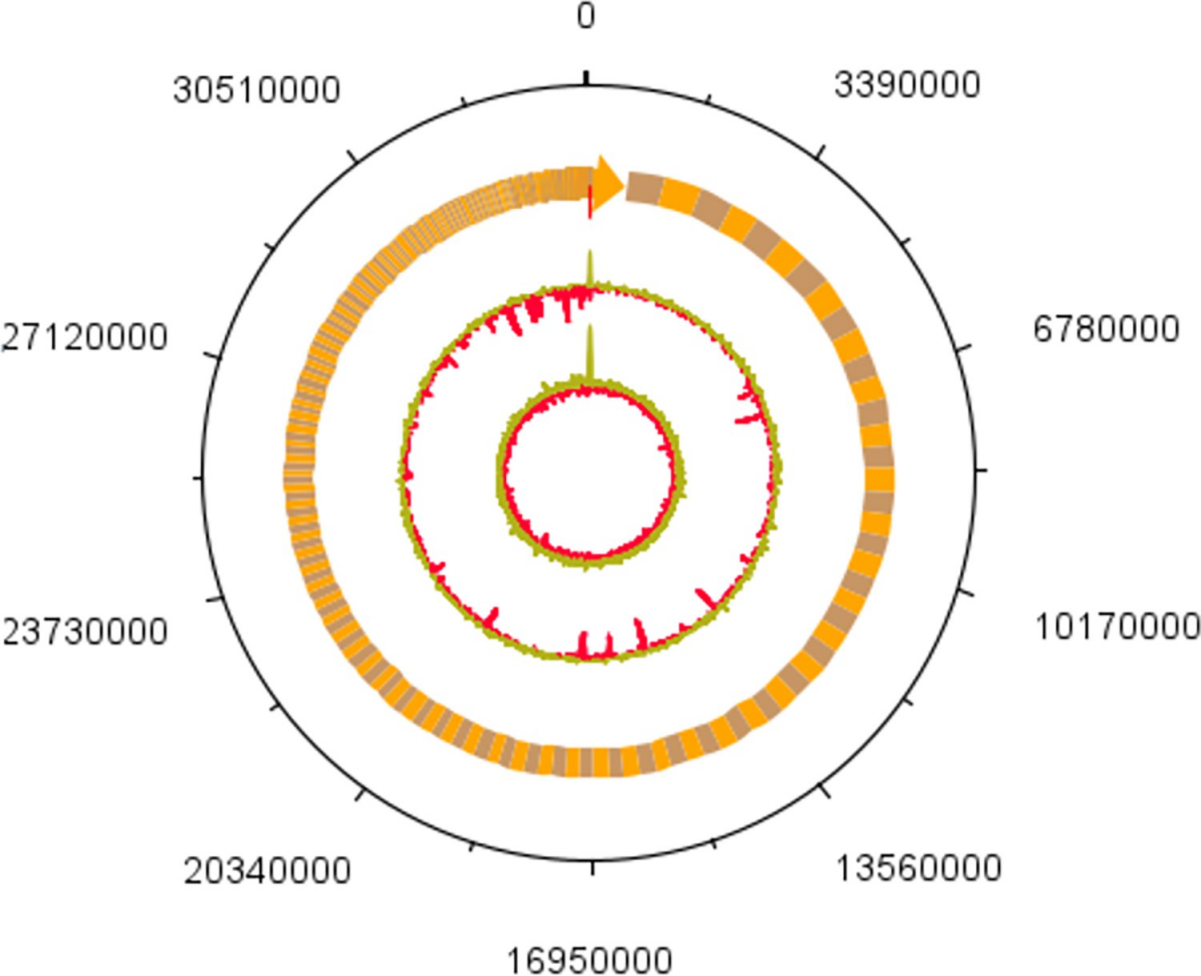
Genome diagram of strain FM, (from inside to outside: first circle shows GC skew, green is the above average of GC skew, red is the below average part of GC skew; the second circle shows the GC content; the third circle shows contigs).

llumina sequencing achieved more than 85x coverage for the genome, yielding a total of 10,193,500 clean reads with Q20 quality at 98.06% and Q30 quality at 94.41%. The GC content of the clean reads was 51.68%. We obtained a draft genome assembly of 33853152 bp in size and organized into 779 contigs. Genome functional annotation was performed with Uniport and KEGG. FM strain had 23107 unique genes resulting in over 49% of genes being protein coding genes annotated with GO term (Fig 1). The whole genome of *E*. *spinifera* strain FM has been deposited in the NCBI database under the accession number ASM3795415v1.

Through whole-genome sequencing and BLAST analysis, we have gained valuable insights into the xenobiotic degradation pathways present in *E*. *spinifera* FM. The BLAST analysis, based on the KEGG database, revealed significant matches to known xenobiotic degradation enzymes (S2 File). The genome analysis of the FM strain uncovered several enzymes involved in xenobiotic degradation pathways, underscoring their particular potential. Notably, we identified the presence of genes encoding enzymes associated with Benzoate, Aminobenzoate, Fluorobenzoate, Chlorobenzene, Toluene, Polyethylene terephthalate (PET), Melamine, Ethylbenzene, and Atrazine degradation pathways in the *E*. *spinifera* FM strain. These findings strongly suggest the strain’s ability to degrade a wide range of xenobiotic compounds. Furthermore, through experimental validation, it has been demonstrated that the *E*. *spinifera* FM strain is capable of degrading Dibenzothiophene [14]. However, during the genome analysis, the specific genes responsible for the enzymes involved in DBT degradation was identified (Table 1) [6, 34–38]. In addition, the genome analysis revealed the presence of all the necessary genes related to enzymes involved in the degradation of other polycyclic hydrocarbons, including Fluorine and Phenanthrene. These pathways involve key reactions, such as hydroxylation, oxidation, conjugation, and ring cleavage, which collectively contribute to the breakdown and detoxification of various pollutants.

**Table 1:** Genes responsible for the enzymes involved in DBT degradation were identified by BLAST analysis.

Genes	FM Current Gene Annotaion	GeneBank no.	FM query	E-value	Bitscore
***DszD***	Flavin_Reduct domain-containing protein	KIW10325	g2	10^−11^	47.4
***DszA***	Bac_luciferase domain-containing protein	KIW13470	g412	10^−76^	235
***DszA***	Bac_luciferase domain-containing protein	KIW09912	g1946	10^−50^	162
***DszA***	Bac_luciferase domain-containing protein	KIW10644	g4496	10^−66^	203
***DszA***	Bac_luciferase domain-containing protein	KIW13111	g4850	10^−78^	239
***DszA***	Bac_luciferase domain-containing protein	KIW14971	g7689	10^−67^	208
***DszA***	Bac_luciferase domain-containing protein	KIW17775	g8127	10^−9^	45.1
***DszA***	Dimethyl sulfone monooxygenase SfnG	KIW15895	g9837	10^−10^	47.0
***DszC***	Uncharacterized protein	KIW15893	9836	10^−41^	133
***DszB***	Uncharacterized protein(Thioesterase)	KIW13453	g430	10^−20^	72.8
***DszB***	Carrier domain-containing protein	KIW19939	g3175	10^−21^	68.6

**Fig 2:**
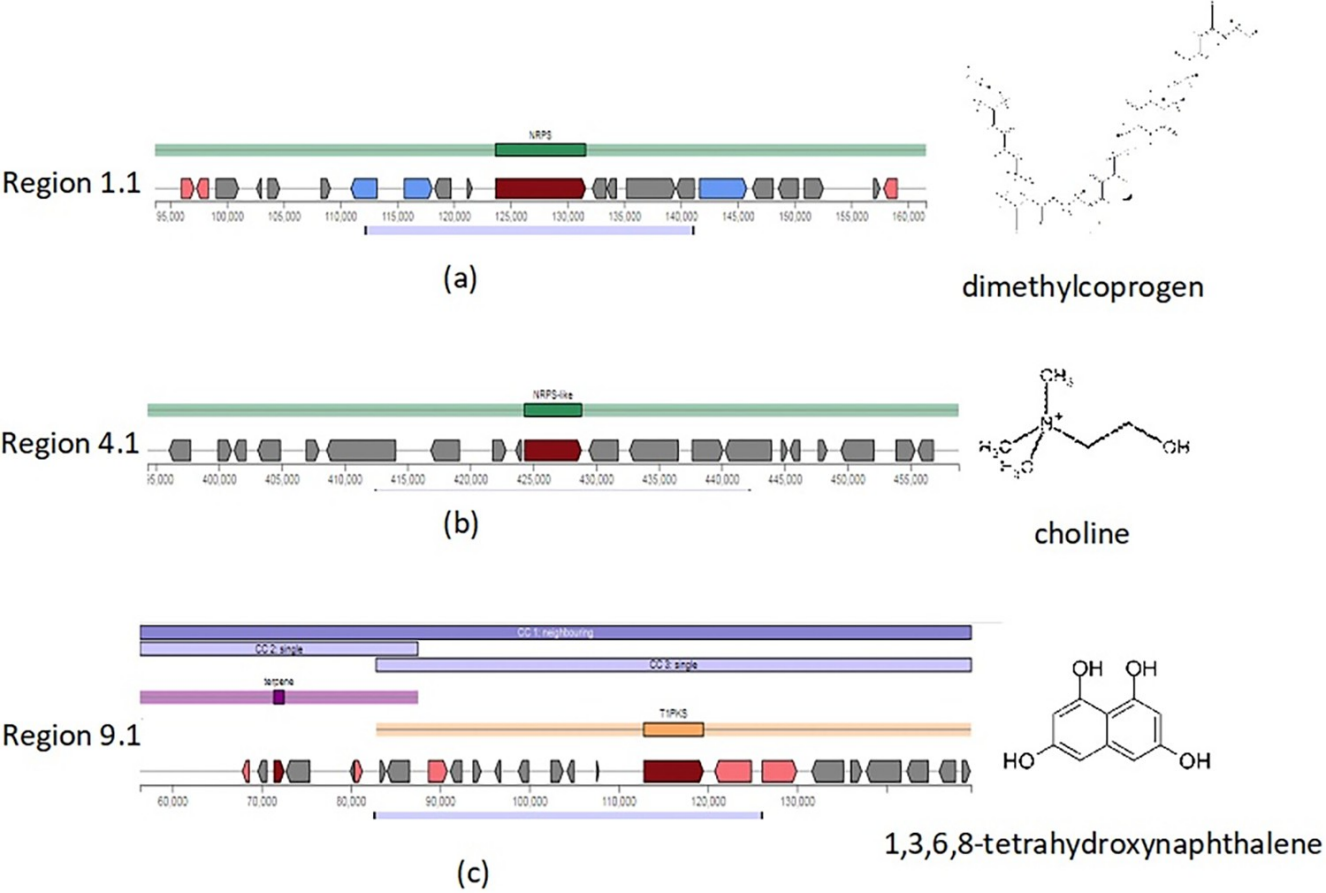
BGCs with 100% identity in strain FM responsible for biosynthesis of (a) Dimethylcoprogen (b) Choline (c) 1,3,6,8-tetrahydroxynaphthalene.

The AntiSMASH analysis revealed that strain FM exhibits a rich biosynthetic capacity, with the presence of 15 biosynthetic gene clusters (BGCs) categorized into eight cluster types. These cluster types include non-ribosomal peptides synthase (NRPS), NRPS-like, Beta-lactone, type I, III polyketides synthase (T1PKS, T3PKS), non-alpha poly-amino acid (NAPAA), Terpene, and Fungal-RiPP-like (Table 2). This finding highlights the significant biosynthetic potential of this environmental strain, indicating its ability to potentially produce a diverse range of secondary metabolites (Fig 2).

**Table 2:** 15 biosynthetic gene clusters (BGCs) found by AntiSMASH analysis.

Region	Location	Total length	Most similar known cluster	Cluster type
**1.1**	93,664–161,596	67,933 nt	Dimethylcoprogen(100%)	NRPS
**4.1**	394,319–458,829	64,511 nt	Choline(100%)	NRPS-like
**8.1**	129,115–164,860	35,746 nt	Oryzine A, oryzine B(48%)	Betalactone
**9.1**	56,378–149,498	93,121 nt	1,3,6,8-tetrahydroxynaphthalene(100%)	T1PKS,Terpene
**11.1**	248,564–337,962	89,399 nt	Phomoidride(67%)	T1PKS
**16.1**	114,063–178,112	64,050 nt	Sesterfisherol(65%)	T3PKS
**29.1**	166,241–237,328	71,088 nt	Prolipyrone B, gibepyrone D(63%)	T1PKS
**30.1**	275,724–309,889	34,166 nt	Carotenoid(44%)	Terpene
**35.1**	1–45,618	45,618 nt	ε-Poly-L-lysine(48%)	NAPAA
**36.1**	170,157–233,608	63,452 nt	Zealexin B2, zealexin C2(67%)	NRPS-like
**37.1**	101,168–132,630	31,463 nt	Squalestatin S1 (66%)	Terpene
**44.1**	1–52,627	52,627 nt	Acetylaranotin(20%)	NRPS-like
**66.1**	129,055–178,118	49,064 nt	Metachelin C, metachelin A, metachelin A-CE, metachelin B(59%)	NRPS
**74.1**	50,572–124,315	73,744 nt	Enterobactin(61%)	NRPS
**101.1**	53,340–119,587	66,248 nt	Carbapenem MM4550(29%)	Fungal-RiPP-like

### General characteristics of the reconstructed GSMM of *Exophiala spinifera* FM

The final curated model, named iEsp1694, consists 4463 reactions, 1694 genes and 3038 metabolites, compartmentalized into 14 subcellular locations. The model includes 1096 transport reactions, 304 exchanges and remaining 3063 (excluding the biomass reaction) are metabolic reactions. In our model 95% of the metabolic reactions are associated to at least one gene. Of those 1694 genes, 48.64% are monofunctional while the rest of the genes (776) conduct more than one reaction in the model. Taking connectivity as the number of reactions in which, a metabolite participates, the model presents 1336 metabolites participate in two metabolic reactions, 394 in three reactions, and 845 in more than three reactions. Table 3 represents a summary of the GSMMs’ main features and how they compare with fully compartmentalized model yeast8 of *S*. *cerevisiae* and iEde2091, *E*. *dermatitidis* model.

**Table 3:** Characteristics of the reconstructed GSMM of *E*. *spinifera* FM.

	iEsp1694	Yeast8	iEde2091
**Total Genes in Genome**	12,047	6,060	9,391
**Included number of genes**	1694	1150	-
**Total number of reactions**	4463	4058	1661
**Total Metabolites**	3038	2742	1856

### Model evaluation

#### Flux balance analysis

**Fig 3:**
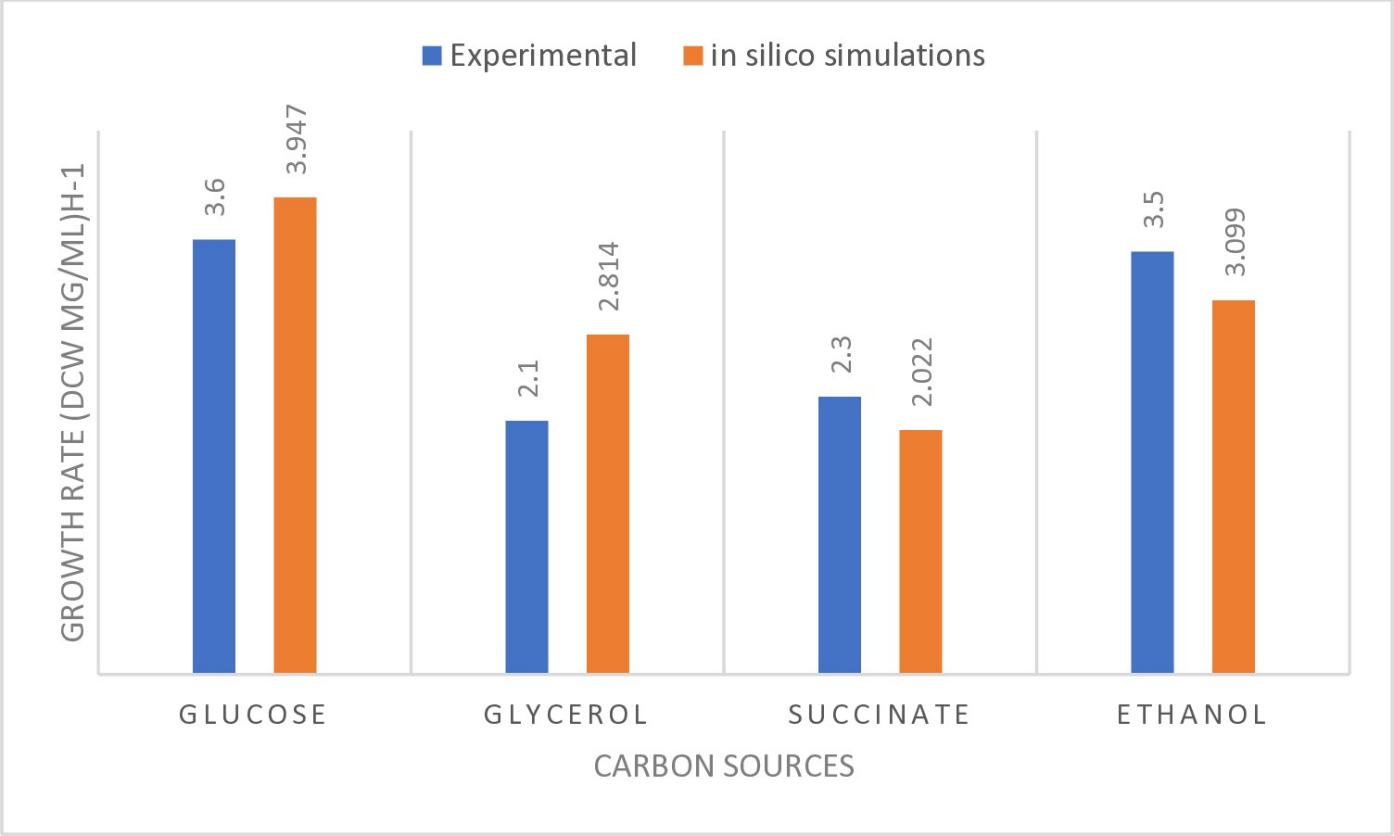
Growth rates at 0.3 mM DBT and different carbon sources.

The primary objective of reconstructing a model is to enable accurate prediction of the physiological behavior of the specific organism. Evaluation of the model was performed using available data on metabolism of *E*. *spinifera* in the scientific literature. To validate the model, we utilized the desulfurization and growth data obtained from the experiments conducted by Elmi et al. [14]. Elmi et al. conducted experiments involving various carbon sources. In our study, we employed iEsp1694 *in silico* model to simulate these experiments and assess cell growth on the four different carbon sources. For each simulation, the carbon content was kept constant for the different carbon sources, while using minimal media to maximize cell growth. Fig 3 presents a comparison of the relative effects of different carbon sources on growth rates, both *in silico* and experimentally. Most of the growth predictions are in agreement with the experimental data.

**Fig 4:**
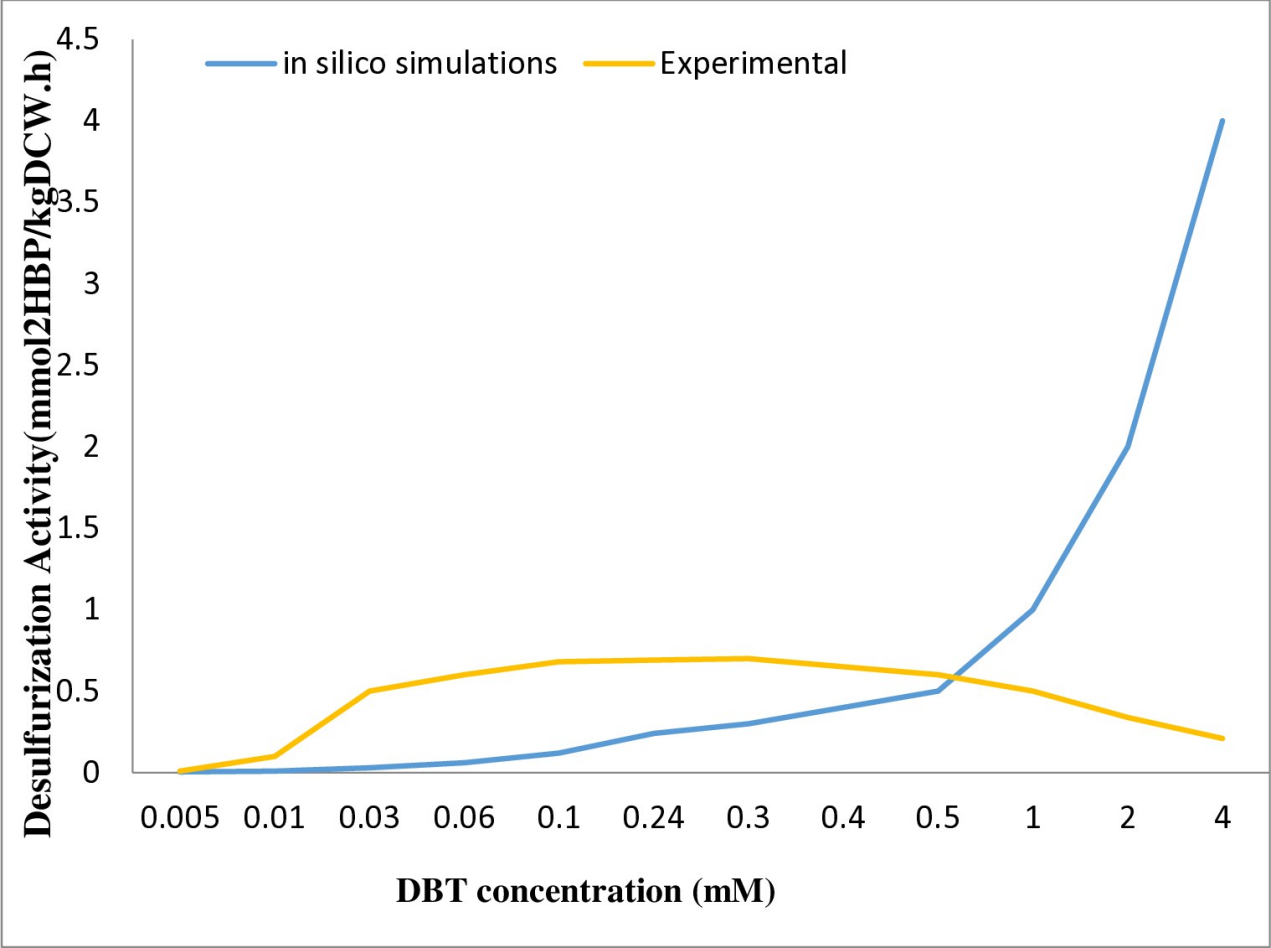
Desulfurization activity (rate of 2-HBP production) fora constant uptake rate of Glucose (1%) and different DBT concentrations.

We further examined the utilization of DBT as a sulfur source at different uptake rates to showcase the robustness of the model in predicting desulfurization activity. In the experiments conducted by Elmi et al. investigated desulfurization activity in the presence of a fixed concentration of glucose (1%) along with varying concentrations of DBT. To investigate this phenotype *in silico*, we set the uptake rate of glucose at a constant rate and analyzed the production of 2-HBP as a final product of desulfurization (Fig 4) with different DBT concentrations. The model simulation analysis confirms the experimental observations to some extent up to 0.5 mM of DBT. However, at higher concentrations of DBT, due to the lack of a regulatory system and the model’s inability to account for the toxic effects of DBT, it cannot accurately predict the behavior of this microorganisms.

Table 4 summarizes the *in vitro* vs *in silico* results. Using both experimental results and available published literature to validate the predictions of iEsp1694, this model was able to correctly predict the phenotypes in 83% of cases.

**Table 4:** Utilization of various carbon and nitrogen sources by *E*. *spinifera* from previously published data and as predicted by the model. A) + (means the compound can be utilized as a sole carbon/nitrogen source while a) − (means that it cannot be.

Carbon and nitrogen sources	*In vitro* results	*In silico* results	Reference
**D-glucose**	+	+	[14, 39, 40]
**D-galactose**	+	+	[39, 40]
**L-sorbose**	+	-	[39, 40]
**D-Glucosamine**	+	+	[39, 40]
**D-Ribose**	+	+	[39, 40]
**D-Xylose**	+	+	[39, 40]
**D-Arabinose**	+	+	[39, 40]
**L-Arabinose**	+	+	[39, 40]
**Ramnose**	+	+	[39, 40]
**Sucrose**	+	+	[39–41]
**Maltose**	+	+	[39, 40]
**α,α-Trehalose**	+	+	[39, 40]
**Cellobiose**	+	+	[39, 40]
**Salicin**	+	-	[39, 40]
**Arbutin**	-	-	[39, 40]
**Melibiose**	-/+	+	[39, 40]
**Lactose**	-	-	[39, 40]
**Raffinose**	+	+	[39, 40]
**Glycerol**	+	+	[39, 40]
**Ribitol**	+	+	[39, 40]
**Xylitol**	+	+	[39, 40]
**Arabinitol**	+	+	[39, 40]
**D-Glucitol**	+	+	[39, 40]
**D-Manitol**	+	+	[39, 40]
**Galactitol**	-	-	[39, 40]
**Inositol**	+	-	[39, 40]
**D-Gluconate**	+	+	[39, 40]
**DL-lactate**	+	+	[39, 40]
**Succinate**	+	+	[14, 39, 40]
**Citrate**	-/+	+	[14, 39, 40]
**Methanol**	-	-	[39, 40]
**Ethanol**	+	+	[14, 39, 40]
**Nitrate**	+	+	[39, 40]
**Nitrite**	+	+	[39, 40]
**Ethylamine**	+	+	[39, 40]
**L-lysine**	+	+	[39, 40]

#### Shadow price analysis

In the context of metabolic modeling, shadow price represents the cost to the objective (growth in this model) to produce one more unit (here mmol/gDW∙h) of a particular metabolite. The analysis is performed using the dual form of the FBA optimization problem to determine the shadow price for each metabolite [42]. Shadow price analysis is a powerful tool for investigating the involvement of metabolites in DBT biodesulfurization pathway of *E*. *spinifera* FM and elucidating the metabolic costs associated with selecting carbon sources. By quantifying the shadow prices, we can better understand of the organism’s overall metabolism. This analysis allows us to assess the impact of different environmental conditions on the metabolic flux distribution and identify key metabolites that play crucial roles in the pathway of interest. This information is valuable for optimizing metabolic engineering strategies and designing more efficient bioprocesses.

The shadow prices serve as indicators of the relative importance of metabolites in the pathways and provide insights into how the organism’s metabolism adapts to different carbon sources and the influence of substrate availability on metabolite production and pathway flux. As we can see in Table 5, the model predicts that under the four carbon sources tested, the 3’-Phospho-5’-Adenylyl Sulfate (PAPS) has a shadow price range of -0.83 to -0.98. This is approximately 8 times more than the shadow price of Dibenzothiophene 5-sulfoxide (DBTO), which has a range of -0.11 to -0.15. Metabolites with higher shadow prices, like PAPS, are likely to be important control points that influence the overall system performance.

**Table 5:** Comparison of shadow prices for metabolites involved in DBT biodesulfurization (mmol2HBP/kgDCW.h) and sulfate assimilatory pathways across different growth conditions (glucose, ethanol, glycerol, and succinate).

Metabolite ids	Metabolite Names	Ethanol	Glycerol	Succinate	Glucose
**s_5154[c]**	Dibenzothiophene 5-sulfoxide (DBTO)	-0.115118633	-0.125459883	-0.12027581	-0.15534812
**s_5155[c]**	Dibenzothiophene 5-sulfone (DBTO2)	-0.230237266	-0.250919765	-0.24055162	-0.31069624
**s_5156[c]**	2-(2’-hydroxyphenyl)benzenesulfinate (HBPS)	-0.345355899	-0.376379648	-0.360827429	-0.46604436
**s_5157[c]**	2-hydroxybiphenyl (2-HBP)	0.115118633	0.125459883	0.12027581	0.15534812
**s_1469[c]**	Sulfite	-0.479458475	-0.522528826	-0.50093764	-0.63929633
**s_1467[c]**	Sulfate	-0.546509763	-0.595603414	-0.570992746	-0.714492497
**s_0717[c]**	FMNH2	-0.537878397	-0.462060914	-0.509082968	-0.440434694
**s_0841[c]**	Hydrogen Sulfide	0.018983943	0.002298811	0.011019112	-0.00477436
**s_0298[c]**	5’-Adenylyl Sulfate (APS)	-0.824940933	-0.866863055	-0.85308201	-0.978275878
**s_0201[c]**	3’-Phospho-5’-Adenylyl Sulfate (PAPS)	-0.831268914	-0.873759487	-0.859693477	-0.989018187
**s_0390[c]**	Adenosine 3’,5’-bismonophosphate	-0.297415113	-0.298845367	-0.308535132	-0.292429539

**Fig 5:**
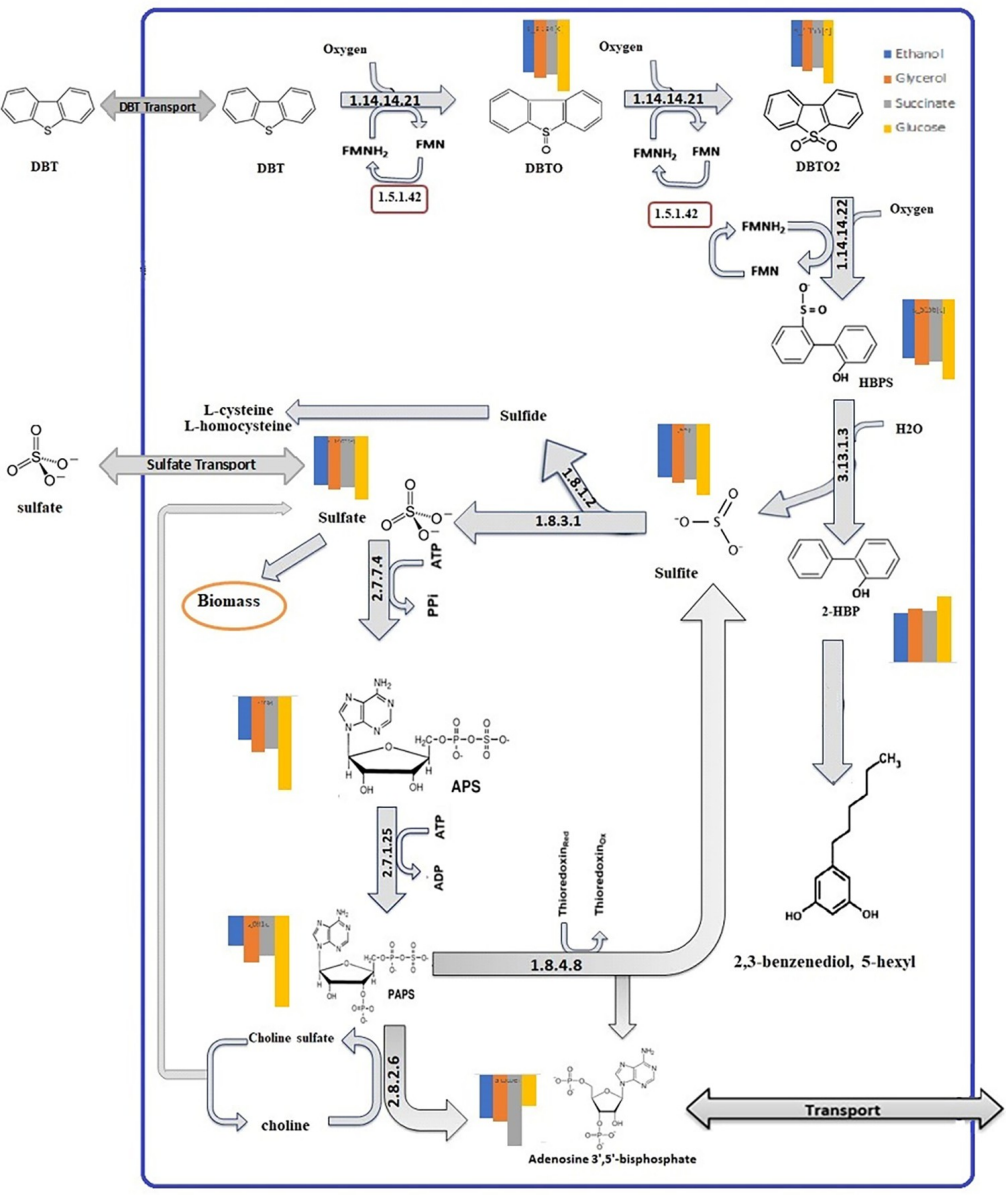
Interplay between DBT biodesulfurization pathway and sulfate assimilatory pathway, with a comparison of shadow prices for intermediate metabolites involved in DBT and sulfate pathways across different carbon sources (glucose, ethanol, glycerol, and succinate).

To gain a comprehensive understanding of the metabolic network, it is crucial to consider the interconnected nature of pathways. Therefore, a thorough analysis should explore how the shadow prices of metabolites in the DBT biodesulfurization pathway relate to other pathways involved in sulfur metabolism or general cellular metabolism. This investigation can identify key metabolites that act as intermediates between pathways and help assess their regulatory roles. Fig 5 illustrates the incorporation of the DBT biodesulfurization pathway and the sulfate assimilatory pathway, with a comparison of shadow prices for metabolites involved in DBT biodesulfurization and sulfate assimilatory pathways across different growth conditions (glucose, ethanol, glycerol, and succinate). Notably, Fig 5 highlights that sulfite is a common intermediate in the microbial metabolism of sulfur-containing compounds. It can be generated through the oxidation of sulfide or the desulfurization of sulfur-containing compounds such as DBT.

An intriguing result emerged regarding the shadow price of 2-hydroxybiphenyl. Unlike most other intermediate metabolites, which displayed negative shadow prices, the shadow price associated with 2-hydroxybiphenyl was positive. When applying shadow price analysis to the model, the majority of shadow price values should be negative, indicating that producing extra of any metabolite detracts carbon from biomass production. It may be the case that some compounds have positive shadow prices indicating that if more is made then more biomass is also made. These issues are likely indicators of mass or charge imbalance in some reaction involving that particular metabolite, or a metabolite upstream of that metabolite in the reaction network. This cannot be true as we have performed manual curation where we ensured that the reactions are both mass and charge balanced.

In the shadow price analysis, results revealed that not all carbon sources are equally effective in the DBT biodesulfurization. Specifically, the shadow costs associated with glucose exhibited higher negative values compared to the other carbon sources. Intermediate metabolites involved in the DBT degradation pathway are generally expensive to produce when grown on glucose. Additionally, the results show that Adenosine 3’,5’-bismonophosphate is more expensive to produce when DBT is degraded in the presence of these carbon sources (glucose, ethanol, glycerol, and succinate). Overall shadow costs of metabolites involved sulfate assimilatory pathway in the presence of all carbon sources, are approximately two times higher than those in the DBT pathway. This indicates that suppressing this pathway when DBT is the sole sulfur source could potentially enhance the performance of the DBT desulfurization pathway.

**Fig 6:**
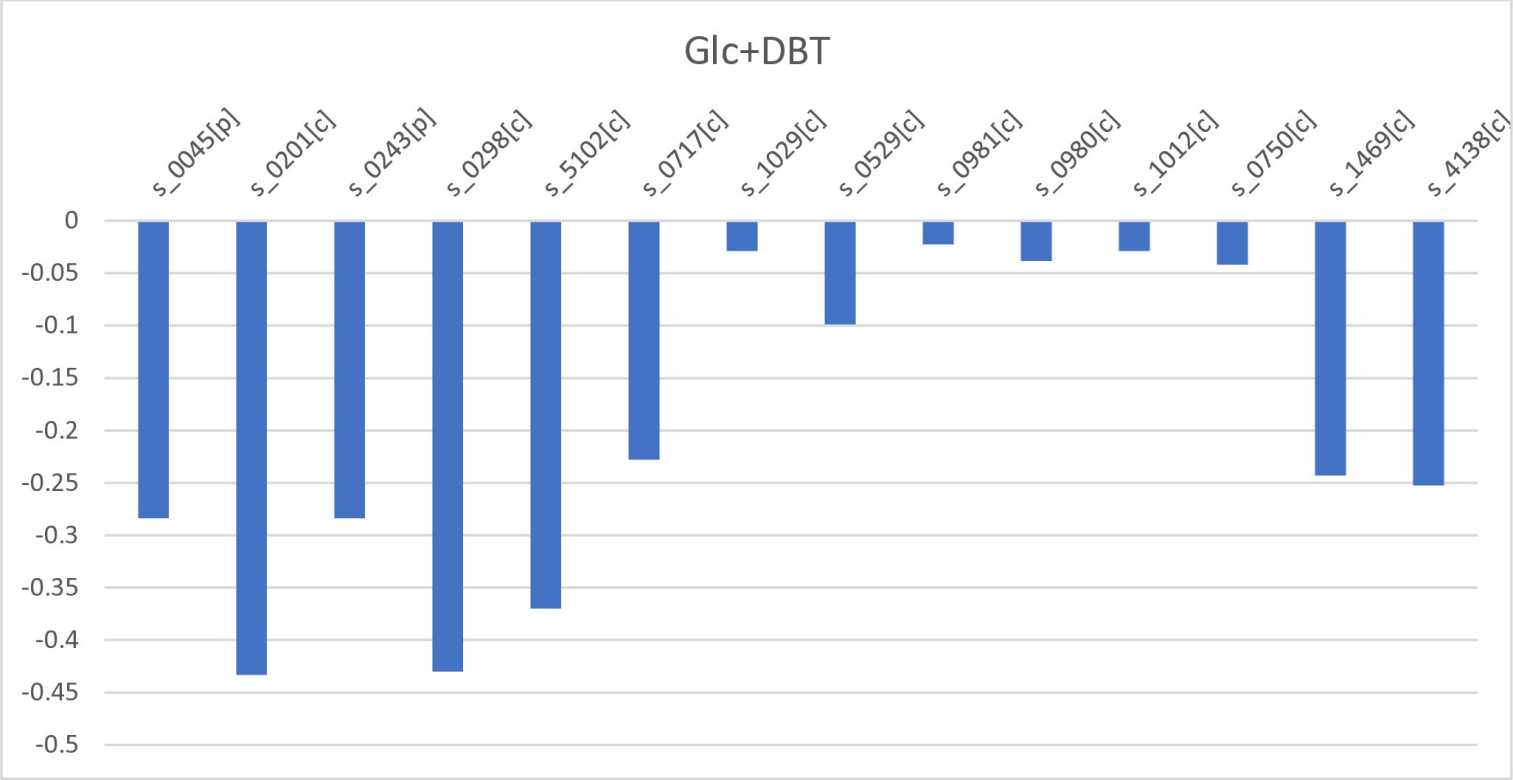
Shadow prices of sulfur-containing metabolites for a constant glucose uptake rate and DBT as the sole sulfur source s_1469[c] = sulfite, s_4138[c] = L-cysteate,s_0750[c] = glutathione, s_1012[c] = L-homocysteine, s_0980[c] = L-cystathionine, s_0981[c] = L-cysteine, s_0529[c] = coenzyme A_c, s_1029[c] = L-methionine, s_0717[c] = FMNH2, s_5102[c] = choline sulfate_c, s_0298[c] = 5’-adenylyl sulfate_c, s_0243[p] = 3-oxohexacosanoyl-CoA_p, s_0201[c] = 3’-phospho-5’-adenylyl sulfate_c, s_0045[p] = (S)-3-hydroxyhexacosanoyl-CoA_p.

For further investigation, we analyzed the shadow prices of sulfur-containing metabolites within the metabolic model (Fig 6). When DBT is used as the sulfur source and glucose is the constant carbon source, we observed that 3’-phospho-5’-adenylyl sulfate, 5’-adenylyl sulfate, and choline sulfate exhibited the most negative shadow prices. This information indicates that these three sulfur-containing metabolites are expensive to produce when DBT is the sulfur source.

### Gene essentiality

Gene essentiality analysis is a critical step in validating the model and gaining insights into fundamental cellular metabolism. In our study, we employed Bidirectional Blast to identify 25,822 orthologous genes in *E*. *spinifera* FM. Among these genes, 1,215 were present in the model and also found in the essentiality datasets of *S*. *cerevisiae*. It is worth noting that many of the genes orthologous to those in *S*. *cerevisiae* were not included in the GSMMs due to their involvement in cellular and genetic information processing functions.

Using Cobrapy [43], we performed single gene knockout simulations in the model to predict a list of growth-essential genes. Among these genes, eight were identified as essential in the model, contradicting the essentiality datasets (False Negative). This discrepancy may be attributed to the absence of alternative genes or pathways in the model, resulting in the *in silico* essentiality of certain genes that are non-essential *in vitro*. Furthermore, our model classified 105 genes as non-essential, while the essentiality data indicated their essentiality (False Positive) (Table 6). This can be partly explained by the use of rich media in both gene essentiality datasets. When simulating gene deletions in rich media, we open all exchange reactions in the model to mimic an experimental setup where all metabolites are present in the growth media. However, if a metabolite is absent from the experimental growth media, a gene may be deemed essential even though it would not be essential if the metabolite were present.

**Table 6:** Gene essentiality analysis and validation. Gene essentiality prediction was correct for 78.1% of tested genes. E = Essential, NE = Non-Essential.

		*In silico*	
		E	NE
**SGD database**	**E**	18 True negative	105 False positive
	**NE**	8 False negative	***931*** True positive

## Discussion

The complete genome sequence of *Exophiala spinifera* FM provides valuable insights into the metabolic potential of a novel hydrocarbon-oxidizing, dibenzothiophene-desulfurizing strain of the genus *Exophiala*. In this study, we obtained a high-quality whole-genome sequence of *E*. *spinifera* and conducted extensive gene prediction and annotation analysis. Through these analyses, we identified new genome annotations that fill gaps in our understanding of the organism’s functionalities. While the confirmation of 2-HBP production and 4S pathway intermediates [44–46] by HPLC analysis in *E*. *spinifera* FM [14] provides some clarity regarding the involvement of the 4S pathway, the current understanding of this process lacks genetic evidence to support the claim. Nevertheless, it is worth noting that we have identified genome annotations associated with enzymes involved in the DBT biodesulfurization pathway, which suggests potential avenues for further investigation. Although the utilization of the 4S pathway encoded by the *dszABC* operon-like structure for sulfur assimilation from DBT in *E*. *spinifera* has not been definitively established, our analysis revealed that the genes *DszA*, *DszB*, *DszD*, and *DszC* showed significant similarity to genes documented in KEGG database [34–38]. This suggests a potential involvement of these genes in sulfur assimilation processes. However, we believe it is crucial to conduct further investigations and experimental analysis to ensure the accuracy of this gene annotation and verify its role in the DBT biodesulfurization pathway. Identifying the first DszB enzyme in fungi would be a significant discovery and could potentially provide insights for metabolic engineering approaches aimed at enhancing biodesulfurization rates [7].

Moreover, the discovery of xenobiotic degradation pathways in *E*. *spinifera* FM holds significant implications for environmental and industrial applications. The strain’s ability to metabolize specific xenobiotic compounds such as Polyethylene terephthalate (PET), Benzoate, Cholorobenzoate, Aminobenzoate, Fluorobenzoate, Ethylbenzene, Toluene, phenanthrene, Atrazine and melamine suggests potential applications in bioremediation [47–51], where it can be employed to mitigate environmental pollution caused by these compounds. Furthermore, the identified pathways may find utility in biotechnological processes, such as the biosynthesis of valuable compounds from xenobiotic precursors, contributing to the development of sustainable and environmentally friendly production strategies [52].

Additionally, AntiSMASH analysis of strain FM revealed that three out of fifteen biosynthetic gene clusters (BGCs) showed 100% similarity with known gene clusters. This suggests that *Exophiala spinifera* FM has the potential to produce other secondary metabolites. Notably, in a commercial oil biodesulfurization process, cost-effective treatment of large volumes of oil is crucial [53]. However, the potential of biodesulfurization biocatalysts extends beyond their application in desulfurization alone. They can also be employed for the production of valuable products, such as the detoxification of chemical warfare agents, as well as the synthesis of surfactants, antibiotics, polythioesters and a range of specialty chemicals. This diversification of applications not only enhance the economic viability of the process but also expands its potential for the production of higher value products with various industrial and commercial uses [52].

We have developed the first GSMM for *E*. *spinifera* FM, which provides a comprehensive framework to understand its metabolic capabilities and offers a computational platform for investigating these significant abilities. iEsp1694 successfully predicts and explains the experimental observations reported in the literature [14, 39, 40, 54].

The shadow price analysis conducted in this study indicates that the production of intermediate metabolites during DBT degradation is more costly when glucose is the sole carbon source which is in agreement with experimental results [14]. These findings have practical implications for optimizing microbial biodesulfurization processes and understanding the metabolic strategies of organisms in the presence of different carbon sources. While investigating the shadow price analysis of sulfur-containing metabolites, we observed metabolites such as 3’-phospho-5’-adenylyl sulfate and 5’-adenylyl sulfate are more expensive when DBT is the sulfur source (Fig 6). This finding is consistent with the observations in Fig 5, which demonstrate that the pathway to produce these metabolites in the presence of DBT is considerably long and energy-consuming. Based on this, one of the model-driven suggestions for enhancing desulfurization activity is to incorporate Adenosine-3’,5’-bisphosphate into the growth medium while simultaneously knocking down enzymes 2.7.7.4 or 2.7.1.25. This approach has been proposed based on the understanding that Adenosine-3’,5’-bisphosphate is the sole essential product of the sulfate uptake pathway. Since there are predicted transporter in the annotated genome that could mediate the uptake of Adenosine-3’,5’-bisphosphate (g10904 in S2 File), by providing this metabolite in the medium and reducing the activity of sulfate uptake pathway enzymes, it is hypothesized that cell growth and desulfurization activity can be significantly enhanced.

Our findings indicate that a significant proportion of gene deletions in iEsp1694 yield the expected outcomes. Specifically, the gene essentiality prediction was accurate for 78.1% of the tested genes, which validates the effectiveness of metabolic modeling in this context. However, despite the relatively high success rate, discrepancies between the model predictions and the experimental data still persist. It is important to note that the experimental results were obtained from a different yeast species, *S*. *cerevisiae*, which could contribute to the observed differences. Additionally, when interpreting these disparities, it is crucial to consider the methodology used to obtain the essentiality data and the inherent variations that exist between different species. These factors can contribute to the observed differences and should be taken into account when evaluating the results and concluding them.

In conclusion, iEsp1694 provides a reasonable confirmation of the experimental observations and effectively captures the inter-relationships among the diverse metabolic activities within *E*. *spinifera*. This enables us to explore additional properties of its metabolic network and develop metabolic engineering strategies to obtain enhanced strains.

## Materials and methods

### DNA isolation and genome sequencing

The FM strain was isolated and purified from oil contaminated soil samples collected from different regions of Iran [14].To prepare DNA for genome sequencing, fungal mycelia were harvested from fresh culture on Sabouraud Dextrose Agar (SDA), and Genomic DNA was extracted following DNA Extraction Protocol [55]. The extracted DNA of *E*. *spinifera* FM was sequenced by Illumina 150-bp paired-end sequencing (genewiz, United States).

### Genome analysis

The raw Illumina reads were examined by using FASTQC v0.11.8 and followed by adaptor removal and quality-based trimming performed with TRIMMOMATIC v0.36 [56]. High-quality reads were assembled by SPAdes v3.10.0 [57]. BUSCO v3 [58] was used to assess the completeness of the final assembled genomes. The first step in building a metabolic model of an organism is to identify all the genes present in that organism [59]. Hence, AUGUSTUS, state-of-the-art software, was used to perform functional annotation and predict protein coding sequences [60]. To enhance the annotations even further, Pathway analysis and Basic Local Alignment Search Tool (BLAST) was performed using a variety of databases, including the Kyoto Encyclopedia of Genes and Genomes (KEGG) database [26], NCBI and UniProt [61] to complete functional annotation of all predicted genes and the resulting metabolic pathways were integrated into the genome-based metabolic model.

Biosynthetic gene clusters for the synthesis of secondary metabolites were predicted using antiSMASH version 6.1.1 (https://antismash.secondarymetabolites.org/, v.6.1.1, accessed on 10 December 2022). AntiSMASH can accurately identify all known secondary metabolic gene clusters when it can use specific profile hidden Markov models [62]. Overall, this comprehensive bioinformatics analysis provided a detailed understanding of the genetic makeup and metabolic capabilities of the FM strain, and served as a foundation for the reconstruction of the first metabolic model for this organism.

### Reconstruction of the model

The RAVEN Toolbox 2.0 [63], an open-source MATLAB package, was utilized to generate a draft model. The *getModelFromHomology* function was executed using the Yeast8 GSMM of *S*. *cerevisiae* [21] as a template model. This choice was based on the close phylogenetic relationship between *E*. *spinifera* and *S*. *cerevisiae* [64] and Yeast8 has exceptional annotation accuracy, particularly in the area of fatty acid metabolism. Additionally, the model provided an extensive amount of information regarding its metabolites and genes, further justifying its selection. Consequently, a compartmentalized draft model was successfully constructed.

Furthermore, the first draft model contained gaps due to incorrect annotation in the template model and lacked reactions in parts of metabolism that were unique to *E*. *spinifera*. Furthermore the *gapfilling* function of Cobrapy [43] was utilized to improve the network. This function suggests reactions that were not included based on the template. Extensive manual curation was also undertaken pathway-by-pathway, involving aligning reactions to KEGG databases and the subsequent addition of these reactions to the model. *E*. *spinifera* is a species of highly melanized black fungus and there is currently limited information available regarding its melanin and carotenoid metabolic processes. In order to gain insights into these pathways, we have adapted the melanins (DHN-melanin, eumelanin and pyomelanin) and carotenoid biosynthesis pathways from *Exophiala dermatitidis* [12], a closely related species with more established knowledge in this area.

The subcellular localization of reactions was refined based on the information from the databases UniProt and SGD [65]. Information pertaining to transport proteins was sourced from TransportDB [66]. Some reactions were defined manually to best fit the biochemistry of *E*. *spinifera*. Biodegradation of DBT is the best examples of these kinds of reactions. The mechanism of DBT biodesulfurization was based on the pathway proposed by Elmi et al. [14].

### Biomass composition

The biomass composition of microorganisms is a highly intricate and dynamic system that is influenced by various factors. In order to accurately simulate the growth of *E*. *Spinifera* using a GSMM, it is crucial to incorporate a biomass reaction that encompasses the essential constituents and macromolecular components of cells or organisms. These components typically include carbohydrates, proteins, lipids, DNA, and RNA, with ATP consumption playing a dominant role [30]. However, due to the lack of available quantitative information regarding the biomass constituents of *E*. *Spinifera* in the current literature, we have adapted the lipid, protein, carbohydrates, DNA and RNA composition in the biomass equation from the most recent GSMM of the ascomycete fungus, *S*. *cerevisiae* with some manual adjustments [21]. Additionally, we have integrated the contributions of carotenoids and melanins from the *E*. *dermatitidis* model [12]. Such adaptation from related organisms is an established practice in the reconstruction of metabolic models [67]. This approach allows us to approximate the biomass composition of *E*. *Spinifera*. Furthermore, we have included specific properties in the biomass composition of desulfurizing microorganisms, such as precursors containing sulfur or involved in sulfur metabolism, as well as higher levels of sulfur-containing amino acids, nucleotides, and cofactors [16]. These adjustments appropriately reflect the sulfur requirements and adaptations to the desulfurization process. This allows for a more comprehensive understanding of *E*. *Spinifera* growth and metabolic capabilities in the context of sulfur metabolism and desulfurization processes.

The stoichiometric ratio of these pseudometabolites in the biomass reaction was determined by using the solver tool in Microsoft Excel whose objective was a biomass molecular weight of 1,000 g/mol [42]. However, it is important to acknowledge that the accuracy of the model predictions may be affected by the assumption of using a biomass equation from a different organism. Therefore, further experimental validation is highly recommended to enhance the reliability of the model.

### Model evaluation

#### Constraints-based flux analysis and simulations

For analyzing and predicting phenotypes, the constraint-based GSMMs typically solve the linear optimization problem Flux Balance Analysis (FBA) [68], a widely used computational method, is based on psudo-steady-state assumption, the concentrations of the cellular metabolites remains constant during the analysis [23]. FBA finds a metabolic flux distribution in steady-state that maximizes a defined objective, e. g., biomass production rate, *V*_*biomass*_. Briefly, FBA is formulated as a linear programming problem;

Maximize Z = *V*_*biomass*_

Subject to *S*.*v* = 0

*V*_*i*,*min*_ ≤ *V*_*i*_ ≤ *V*_*i*,*max*_ (for i = 1, …., n)

Where Z denotes the objective function, C is a row vector showing the influence of individual fluxes on the objective function, v is the reaction flux vector, n denotes the number of reactions. *V*_*i*,*min*_ and *V*_*i*,*max*_ are the lower and upper bounds of the flux *V*_*i*_ [23, 68].

Gurobi was used as the optimization solver for the FBA analysis. The model refinement process involved simulating various growth conditions, including the utilization of different carbon sources such as ethanol, glucose, and succinate. All simulations pertaining to gene essentiality, utilization of carbon sources were conducted by FBA. The *optimizeCbModel* function was employed to execute FBA for these simulations. For all metabolites present in the media, the upper and lower bounds of its Exchange reaction were set to -1 and 1 mmol/g/h, respectively. The model can be further constrained by setting fixed fluxes for one or more extracellular metabolites, which are determined based on experimentally measured uptake or release rates. By FBA analysis, we can obtain potential flux distributions that reflect the metabolic state of a cell under specific environmental conditions. To pursuit this goal, a cellular objective function is required, such as maximizing cell growth, minimizing substrate utilization, or minimizing maintenance energy [16]. Among these objectives, cell growth is the most commonly used, as microbial cells have evolved to optimize growth. After the refinement of the metabolic network, the GSMM needed to be further evaluated for the accurate simulation of the metabolic process for some important precursors and synthesis of biomass. Evaluation of the model was performed based on available data on metabolism of *E*. *spinifera* in the experimental literature [14]. Subsequently, for each case, exchange reactions were defined to simulate the growth conditions. S1 File, shows the media content used in the growth simulations for carbon and sulfur sources.

The predictions of the model were then compared to experimental observations. Inconsistencies were checked manually and corrected for adding, removing or changing corresponding reactions whenever possible. Experimental values were used for all simulations. We sequentially opened the exchange reaction for the carbon sources and tested for growth of the model.

### Shadow price analysis

Shadow price analysis, which is the dual formulation of FBA [69], is a technique in linear programming and optimization that helps assess the importance or marginal value of constraints or variables in a metabolic model. Specifically, it quantifies the impact of producing one additional unit (mmol.gD *W*^−1^.*h*^−1^) of a metabolite on the objective function used in FBA (growth). The shadow price associated with variable *i* is defined as the reduction in the optimization objective caused by producing one more unit of *i* [12]. In the COBRApy [43], shadow prices and reduced costs can be calculated by *optimizeCbModel*. Flux balance analysis (FBA) problem maximizing the biomass production was solved, and then shadow prices of metabolites were calculated by *shadow_price* function.

The model was subjected to condition with glucose as the carbon source and DBT as thesulfur source. Then, We calculated the shadow prices of compounds that contain sulfur atoms, including methionine, cysteine, coenzyme A (CoA), and CoA-containing metabolites such as malonyl-CoA and acetyl-CoA. By analyzing the per-sulfur atom shadow costs of these sulfur-containing compounds, we aimed to assess the sulfur requirements of *Exophiala spinifera*. Furthermore, we investigated how much biomass would need to be catabolized in order to produce an additional mole of a sulfur-containing compound.

In a separate analysis, we conducted simulations for four different conditions, each based on a specific carbon source (glucose, ethanol, glycerol, or succinate). The carbon content was kept constant for the different carbon sources. The focus of this analysis was to calculate the shadow cost of intermediate metabolites involved in sulfur pathways when DBT served as the sole sulfur source. By assessing the shadow costs of these intermediate metabolites, we aimed to gain insights into the metabolic impact and resource allocation associated with sulfur utilization in the presence of DBT. This analysis contributes to our understanding of sulfur metabolism and its modulation under different carbon sources, providing valuable information for studying sulfur assimilation in relevant biological systems.

### Gene essentiality

The validation of GSMMs through gene essentiality prediction is a valuable approach to assess and enhance the accuracy of predictions while providing a framework to contextualize knockout mutant studies [70]. Gene essentiality analysis in metabolic models involves simulating the effects of gene knockout or knockdown on the metabolic network. This is typically achieved by constraining the model to simulate the behavior of a cell in which a specific gene has been deleted or inactivated [16]. Subsequently, a computational analysis is performed to determine whether the cell can still grow and function normally. A growth rate of zero indicates that the deleted gene is essential for growth under the tested conditions. Conversely, a non-zero growth rate suggests that the deleted gene is non-essential for growth. COBRApy [43] contains a function, which allows all genes in the model to be deleted individually, and for each gene deletion growth is assessed. This function is similar to in vivo experiments of tn-seq and transposon mutagenesis experiments [71]. Since all gene essentiality experiments were carried out in rich media (e.g. Broth), we performed gene essentiality testing by simulating an *in silico* rich media conditions. To mimic these conditions, we opened all exchange reactions in the model, allowing the unrestricted uptake and secretion of metabolites. We employed Cobrapy, to perform gene knockout simulations. Gene removal involves setting the fluxes of all associated reactions to zero. However, if a reaction was controlled by two or more isozymes, then the reaction was kept active in the absence of any one of the associated genes. To assess the robustness of *E*. *spinifera* metabolism, we investigated its ability to exhibit *in silico* growth in the case of gene knockouts or mutations.

Since experimental gene essentiality data for *E*. *spinifera* is unavailable, we employed a comparative approach using NCBI Bidirectional Blast to identify orthologous genes. We compared *E*. *spinifera* with the closely related species *S*. *cervisiae*, utilizing an E-value threshold of 1e-6 to indicate a higher degree of similarity between the query sequence and the *S*. *cerevisiae* sequence. We utilized experimental data on gene essentiality from the *S*. *cerevisiae* database (SGD).

"This study was conducted primarily using computational and bioinformatic analyses, and did not involve any field work or experiments requiring permits. All data used in the analyses were obtained from publicly available databases. As such, no specific permits were required for the completion of this work."

## Supporting information

S1 File All reactions and metabolites involved in the model, also information about biomass. reaction and media content used in the simulations, are included. (XLSX)

S2 File Genome annotation results. (XLSX)

S3 File iEsp1694 model. (XLSX)
